# Interaction between secondary mitral regurgitation and left atrial function and their prognostic implications after cardiac resynchronization therapy

**DOI:** 10.1093/ehjci/jeac149

**Published:** 2022-07-28

**Authors:** Jan Stassen, Xavier Galloo, Kensuke Hirasawa, Pieter van der Bijl, Martin B Leon, Nina Ajmone Marsan, Jeroen J Bax

**Affiliations:** Department of Cardiology, Leiden University Medical Center, Albinusdreef 2, 2300 RC Leiden, The Netherlands; Department of Cardiology, Jessa Hospital, Stadsomvaart 11, 3500 Hasselt, Belgium; Department of Cardiology, Leiden University Medical Center, Albinusdreef 2, 2300 RC Leiden, The Netherlands; Department of Cardiology, Universitair Ziekenhuis Brussel, Laarbeeklaan 101, 1090 Brussels, Belgium; Department of Cardiology, Leiden University Medical Center, Albinusdreef 2, 2300 RC Leiden, The Netherlands; Department of Cardiology, Leiden University Medical Center, Albinusdreef 2, 2300 RC Leiden, The Netherlands; Department of Cardiology, Columbia University Irving Medical Center/Presbyterian Hospital and Cardiovascular Research Foundation, New York, NY 10032, USA; Department of Cardiology, Leiden University Medical Center, Albinusdreef 2, 2300 RC Leiden, The Netherlands; Department of Cardiology, Leiden University Medical Center, Albinusdreef 2, 2300 RC Leiden, The Netherlands; Turku Heart Center, University of Turku and Turku University Hospital, Kiinamyllynkatu 4-8, FI-20520 Turku, Finland

**Keywords:** functional mitral regurgitation, left atrial reservoir strain, heart failure, cardiac resynchronization therapy, mortality

## Abstract

**Aims:**

Left atrial (LA) function is a strong prognostic marker in patients with heart failure and functional mitral regurgitation (MR). Although cardiac resynchronization therapy (CRT) has shown to improve MR severity, the interaction between a reduction in MR severity and an increase in LA function, as well as its association with outcomes, has not been investigated.

**Methods and results:**

LA reservoir strain (RS) was evaluated with speckle tracking echocardiography in patients with at least moderate functional MR undergoing CRT implantation. MR improvement was defined as at least 1 grade improvement in MR severity at 6 months after CRT implantation. The primary endpoint was all-cause mortality. A total of 340 patients (mean age 66 ± 10 years, 73% male) were included, of whom 200 (59%) showed MR improvement at 6 months follow-up. On multivariable analysis, an improvement in MR severity was independently associated with an increase in LARS (odds ratio 1.008; 95% confidence interval 1.003–1.013; *P* = 0.002). After multivariable adjustment, including baseline and follow-up variables, an increase in LARS was significantly associated with lower mortality. MR improvers showing LARS increasement had the lowest mortality rate, whereas outcomes were not significantly different between MR non-improvers and MR improvers showing no LARS increasement (*P* = 0.236).

**Conclusion:**

A significant reduction in MR severity at 6 months after CRT implantation is independently associated with an increase in LARS. In addition, an increase in LARS is independently associated with lower all-cause mortality in patients with heart failure and significant functional MR.

## Graphical Abstract

**Graphical Abstract:**
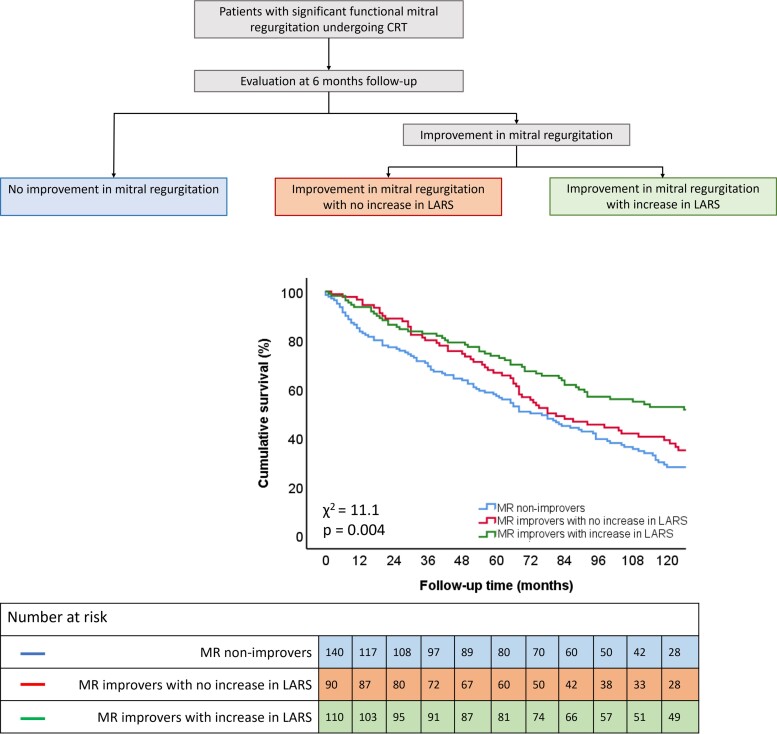

## Introduction

Functional mitral regurgitation (MR) is common in patients with heart failure (HF) and reduced left ventricular (LV) ejection fraction (EF) and is associated with an increased risk of HF hospitalization and mortality.^1,2^ Cardiac adverse remodelling in patients with HF and functional MR does not only affect the LV, but also the left atrium (LA).^3,4^ LA adverse remodelling is accompanied by an increase in interstitial fibrosis of the atrial wall, leading to a reduction in LA compliance. A reduced LA compliance increases LA pressure and the pulsatile load on the pulmonary circulation, contributing to the development of pulmonary hypertension and right ventricular-pulmonary artery uncoupling.^5^ Recent studies have shown that LA reservoir function, measured by speckle-tracking echocardiography, has incremental prognostic value over LA volume and LV systolic function in patients with HF and reduced LVEF, as well as in patients with moderate and severe functional MR.^6,7^

Cardiac resynchronization therapy (CRT) is an established treatment for well-selected patients with HF who remain symptomatic despite optimal medical therapy and has the potential to reduce functional MR severity and induce LA reverse remodelling.^8–10^ Both LA reverse remodelling and a reduction in MR severity have been linked to better cardiovascular outcomes in patients with HF receiving CRT.^9,11,12^ Despite this evidence, the interaction between a reduction in MR severity and an improvement in LA function, as well as their association with outcomes, has not been investigated. Accordingly, the aim of the current study was to investigate the association between a reduction in MR severity and an increase in LA reservoir strain (RS) and their prognostic implications in CRT recipients with moderate or severe functional MR.

## Methods

### Patient population

Patients who had significant (i.e. moderate and severe) functional MR and underwent CRT implantation between July 2000 and September 2014 according to prevailing guideline recommendations^8^ were included from an ongoing single-centre registry at the Leiden University Medical Center in the Netherlands. An ischaemic aetiology of HF was defined by the presence of significant coronary artery disease on invasive coronary angiography. Patients with less than moderate functional MR, previous mitral valve intervention, primary or mixed aetiology, haemodynamically unstable condition at the time of echocardiography or with echocardiographic image quality not allowing accurate two-dimensional speckle tracking echocardiography (i.e. insufficient tracking of the LA wall or the LA could not be entirely visualized), were excluded (see Supplementary data online, *Figure S1*). No patients underwent percutaneous or surgical mitral valve intervention at follow-up. All patients underwent complete clinical and echocardiographic evaluation. Patient information was prospectively collected from the departmental cardiology information system (EPD-vision; Leiden University Medical Center, Leiden, the Netherlands) and retrospectively analyzed. Clinical data included demographic characteristics, cardiovascular risk factors, comorbidities, New York Heart Association functional class and HF medication. Quality of life was evaluated using the Minnesota Living with HF Questionnaire. The study complies with the Declaration of Helsinki and was approved by the Institutional Review Board. Considering the retrospective nature of the study and all data being handled anonymously, the Medical Ethical Committee waived the need for patient written informed consent.

### Echocardiography

All patients underwent transthoracic echocardiography before [median time: 0 (0–0) months] and 6 months [median time: 6 (5–6) months] after CRT implantation in the left lateral decubitus position with commercially available ultrasound equipment (Vivid 7 and E9, GE-Vingmed, Horten, Norway). Electrocardiographic-triggered echocardiographic data were stored digitally in a cine-loop format for offline analysis using EchoPAC version 203 (GE Medical Systems, Horten, Norway). Measurements were performed by two experienced echocardiographers (J.S., X.G.). LV volumes, LVEF and LA volumes were measured using the biplane Simpson’s method.^13^ Tricuspid annular plane systolic excursion was measured on M-mode recordings of the lateral tricuspid annulus in a right ventricular-focused view.^13^ The severity of mitral and tricuspid regurgitation was assessed using a multiparametric approach, as recommended by current guidelines.^14^ MR severity was graded according to current recommendations using an integrative approach that includes qualitative, semiquantitative and quantitative data and was graded on a 4-point scale: none (grade 0), mild (grade 1), moderate (grade 2) and severe (grade 3).^14^ Significant MR was defined as grade 2 or 3. Speckle-tracking LV global longitudinal strain (GLS) was averaged from 17 LV segments, and measured from apical views (two-, three-, and four-chamber).^15^ The region of interest was traced manually and adjusted to the myocardial thickness. LA speckle-tracking strain was measured from the apical four-chamber view with the onset of the QRS complex used as the zero-reference point.^16^ The endocardium of the LA wall was traced manually and corrected by adjusting the region of interest or the width of the contour, excluding the pulmonary vein ostia and LA appendage. LARS was measured directly from the LA strain vs. time curve.^12^ LARS was chosen over LA conduit strain and LA contractile strain because it shows a good correlation with LA wall fibrosis on cardiac magnetic resonance imaging^17^ and can be measured in patients with atrial fibrillation.^16^ Both LV GLS and LARS are represented as absolute (i.e. positive) values.

### CRT implantation

CRT implantation was performed according to a standard approach, i.e. insertion of the right atrial and ventricular leads via the subclavian or cephalic veins. Before insertion of the LV lead, coronary sinus venography was performed. The LV pacing lead was then introduced into the coronary sinus through an 8 Fr guiding catheter, and positioned in a posterior or posterolateral vein, if possible. A posterior or lateral LV lead position was accomplished in 85% of the patients. Defibrillator functionality was present in 98% of the implanted devices. CRT recipients were followed up at regular intervals at the HF outpatient clinic, at which time the device was interrogated. Atrioventricular and interventricular delays were empirically set at 120–140 and 0 ms, respectively. CRT optimization was performed during follow-up visits at the discretion of the treating physician.

### Clinical endpoints

Patients were followed-up for the occurrence of all-cause mortality. Data on mortality were obtained from the departmental cardiology information system (EPD-Vision, Leiden University Medical Center, Leiden, The Netherlands), which is linked to the governmental death registry database. Follow-up data were complete for all patients.

### Statistical analysis

Continuous data are presented as mean ± standard deviation when normally distributed and as median and interquartile range when not normally distributed. Categorical data are presented as frequencies and percentages. Continuous variables were compared using the independent samples Student’s *t*-test when normally distributed, whereas the Mann–Whitney *U* test was used to compare continuous variables that did not have a normal distribution. Categorical variables were compared using the Pearson chi-square test. The inter- and intra-observer variability of LARS measurements were assessed by calculating the intra-class correlation coefficient on 20 randomly selected patients. The intra-class correlation coefficients for inter- and intra-observer variability were 0.94 [95% confidence interval (CI): 0.85–0.97, *P* < 0.001] and 0.95 (95% CI: 0.87–0.98, *P* < 0.001), respectively. The intra-class correlation coefficients for inter- and intra-observer variability of LV GLS were 0.92 (95% CI: 0.84–0.97, *P* < 0.001) and 0.97 (95% CI: 0.89–0.99, *P* < 0.001), respectively. The interobserver variability of grading MR severity was assessed in 50 randomly selected patients, showing excellent interobserver agreement (Cohen’s Kappa 0.96; 95% CI: 0.88-0.99; *P* < 0.001). The association between MR improvement and echocardiographic parameters of cardiac remodelling was evaluated using multivariable logistic regression analysis. The odds ratio (OR) and 95% CI were calculated and reported. Patients were divided in two groups according to the change in functional MR severity at 6 months after CRT implantation: MR improvers and MR non-improvers. MR improvement was defined as an improvement of at least 1 grade in MR severity. Event-free survival curves were generated using the Kaplan–Meier method and differences between groups were analyzed using the log-rank test. Uni-and multivariable analyses of time to event were performed using Cox proportional hazard models. The hazard ratio (HR) and 95% CI were calculated and reported. In the univariable analysis, variables with a *P*-value <0.05 were considered statistically significant and entered in the multivariable model. The proportional hazards assumption was verified through the evaluation of Schoenfeld residuals. To inspect for multicollinearity, the Pearson correlation coefficient was calculated between continuous variables, assuming no significant multicollinearity when the correlation coefficient was <50%. In addition, the Variation Inflation Factor was calculated, assuming no significant multicollinearity when this value was <5. A two-tailed *P*-value <0.05 was considered statistically significant. Statistical analysis was performed using SPSS for Windows, version 25.0 (IBM Corporation, Armonk, New York).

## Results

### Patient population

A total of 340 patients (mean age 66 ± 10 years, 73% male) who had significant functional MR and subsequently underwent CRT implantation were included (see Supplementary data online, *Figure S1*). Of these 340 patients, 235 (69%) had moderate MR and 105 (31%) severe MR. Baseline clinical variables are shown in *Table 1*, while *Table 2* summarizes the echocardiographic data for the overall population prior to CRT implantation. The majority of patients (70%) were in New York Heart Association functional class III-IV and 59% of the patients had an ischaemic aetiology of HF. Mean LVEF was 26 ± 8%, mean LV GLS was 7.3 ± 3.2%, median LARS was 10.5% (7.1–16.7%) and mean vena contracta was 6.0 ± 1.9 mm.

**Table 1:** Baseline clinical characteristics

	Overall population (*N* = 340)	MR non-improvers (*N* = 140)	MR improvers (*N* = 200)	*P*- value
Age, years	66 (±10)	65 (±11)	67 (±9)	0.062
Male sex (%)	249 (73.2%)	105 (75.0%)	144 (72.0%)	0.539
Arterial hypertension (%)	169 (49.7%)	68 (50.4%)	101 (49.2%)	0.840
Diabetes mellitus (%)	65 (19.1%)	29 (20.7%)	36 (18.0%)	0.531
Dyslipidaemia (%)	142 (42.5%)	57 (41.6%)	85 (43.1%)	0.779
Current smoker (%)	48 (14.2%)	18 (13.0%)	30 (15.1%)	0.142
BMI, kg/m^2^	26.1 (±3.9)	25.7 (±3.9)	26.5 (±3.9)	0.064
Ischaemic aetiology (%)	199 (58.5%)	86 (61.4%)	113 (56.5%)	0.364
PCI	25 (7.4%)	14 (10.0%)	11 (5.5%)	0.118
CABG	35 (10.3%)	14 (10.0%)	21 (10.5%)	0.881
QoL score	31 (17–45)	36 (20–51)	26 (14–43)	**0.001**
6MWT, metres	331 (±111)	320 (±108)	340 (±112)	0.138
NYHA III-IV (%)	236 (69.8%)	107 (76.4%)	129 (65.2%)	**0.026**
Sinus rhythm (%)	233 (68.5%)	89 (63.6%)	144 (72.0%)	0.218
QRS duration, ms	157 (±34)	157 (±36)	158 (±33)	0.847
Beta-blocker (%)	250 (73.5%)	101 (72.1%)	149 (74.5%)	0.628
ACE-i/ARB (%)	301 (88.5%)	119 (85.0%)	182 (91.0%)	0.088
MRA (%)	147 (43.2%)	63 (45.0%)	84 (42.0%)	0.583
Diuretics (%)	286 (84.1%)	120 (85.7%)	166 (83.0%)	0.500
Statin (%)	197 (57.9%)	79 (56.4%)	118 (59.0%)	0.636
eGFR, mL/min/1.73 m^2^	63.2 (±22.7)	62.6 (±24.8)	63.7 (±21.2)	0.667
Haemoglobin, g/dL	13.3 (±1.6)	13.0 (±1.6)	13.4 (±1.4)	**0.002**

Values are presented as mean ± SD, median (IQR), or *n* (%). Values in bold have a *P*-value < 0.05.

ACE-i, angiotensin-converting enzyme inhibitor; ARB, angiotensin receptor blocker; BMI, body mass index; CABG, coronary artery bypass grafting; eGFR, estimated glomerular filtration rate; MRA, mineralocorticoid receptor antagonist; MWT, minute walking test; NYHA, New York Heart Association; PCI, percutaneous coronary intervention; QoL, quality of life.

**Table 2:** Baseline echocardiographic characteristics

	Overall population (*N* = 340)	MR non-improvers (*N* = 140)	MR improvers (*N* = 200)	*P*-value
LV EDV, mL	208 (162–254)	206 (157–264)	208 (164–245)	0.837
LV ESV, mL	153 (113–190)	154 (107–203)	153 (117–188)	0.831
LVEF, %	26.4 (±7.7)	26.4 (±8.2)	26.4 (±7.4)	0.995
LV GLS, %	7.3 (±3.2)	7.2 (±3.4)	7.3 (±3.1)	0.629
LAVi, mL/m^2^	46.1 (±17.5)	48.7 (±17.2)	44.3 (±17.5)	**0.025**
LARS, %	10.5 (7.1–16.7)	9.3 (6.3–14.7)	11.6 (7.5–17.6)	**0.016**
TAPSE, mm	16.3 (±4.9)	16.2 (±5.4)	16.4 (±4.5)	0.714
TR max velocity, m/s	2.7 (±0.5)	2.8 (±0.5)	2.7 (±0.6)	**0.041**
Moderate or severe TR (%)	90 (30.9%)	48 (39.0%)	42 (25.0%)	**0.011**

Values are presented as mean ± SD, median (IQR), or *n* (%). Values in bold have a *P*-value < 0.05.

EDV, end-diastolic volume; ESV, end-systolic volume; LAVi, left atrial volume index; TR, tricuspid regurgitation.

There were 200 (59%) patients that showed at least 1 grade improvement in MR severity at 6 months’ follow-up. The percentage of patients showing MR improvement was not different between patients with moderate vs. severe MR at baseline (56% vs. 65%, *P* = 0.094). Baseline vena contracta was not different between MR improvers and MR non-improvers (5.9 ± 1.9 vs. 6.0 ± 2.0 mm; *P* = 0.549). Patients who showed MR improvement were less likely to be in New York Heart Association III-IV, had a higher quality of life score (according to the Minnesota Living with HF Questionnaire) and had higher haemoglobin values at baseline. In addition, patients who showed MR improvement had smaller LA volumes, more preserved LARS, lower tricuspid regurgitation velocities and a lower percentage of concomitant moderate or severe tricuspid regurgitation. There was no difference in LV lead placement in MR improvers vs. MR non-improvers (*P* = 0.103).

### Changes in echocardiographic characteristics at 6 months’ follow-up

Changes in echocardiographic parameters at 6 months’ follow-up, expressed as a percentage change, are summarized in *Table 3*. Changes, expressed as an absolute change, are shown in Supplementary data online, *Table S1*. Patients who showed an improvement in MR severity had a more pronounced reduction in LV end-diastolic volume, LV end-systolic volume and LA volume and a more pronounced increase in LVEF and LARS. Of interest, none of the patients underwent coronary intervention (percutaneous coronary intervention or coronary artery bypass grafting) between baseline and 6 months follow-up.

**Table 3:** Relative changes in echocardiographic variables at 6-months follow-up

	Overall population (*N* = 340)	MR non-improvers (*N* = 140)	MR improvers (*N* = 200)	*P*- value
LV EDV, %	−10 ± 19	−2 ± 18	−16 ± 18	**<0.001**
LV ESV, %	−17 ± 23	−7 ± 22	−24 ± 20	**<0.001**
LVEF, %	31 ± 43	21 ± 40	37 ± 43	**0.001**
LV GLS, %	35 ± 7	24 ± 8	43 ± 10	0.178
LAVi, %	−3 ± 34	3.5 ± 37	−7 ± 30	**0.004**
LARS, %	31 ± 5	10 ± 5	46 ± 8	**<0.001**
TAPSE, %	9 ± 36	11 ± 35	7 ± 37	0.475
TR max velocity, %	6 ± 59	1.4 ± 19	8.9 ± 78	0.337

Values are presented as mean ± SD. Values in bold have a *P*-value < 0.05.

EDV, end-diastolic volume; ESV, end-systolic volume; LAVi, left atrial volume index; TR, tricuspid regurgitation.

### Independent association between MR improvement and changes in echocardiographic characteristics

*Table 4* shows the multivariable logistic regression analysis to assess the association between changes in echocardiographic parameters at 6 months’ follow-up and MR improvement. A reduction in LV end-systolic volume (OR 0.974; 95% CI: 0.957–0.991; *P* = 0.003) and an increase in LARS (OR 1.008; 95% CI: 1.003–1.013; *P* = 0.002) at 6 months after CRT implantation, were independently associated with MR improvement.

**Table 4:** Association between MR improvement and echocardiographic parameters of cardiac remodelling

	Multivariable analysis	
	OR (95% CI)	*P*-value
Relative change in LV ESV	0.974 (0.957–0.991)	**0.003**
Relative change in LVEF	1.006 (0.961–1.053)	0.795
Relative change in LV GLS	0.999 (0.996–1.002)	0.701
Relative change in LAVi	0.997 (0.989–1.005)	0.492
Relative change in LARS	1.008 (1.003–1.013)	**0.002**
Relative change in TR max velocity	1.002 (0.995–1.010)	0.529

Values in bold have a *P*-value < 0.05.

ESV, end-systolic volume; LAVi, left atrial volume index; TR, tricuspid regurgitation.

### LARS and MR improvement: prognostic implications

**Figure 1:**
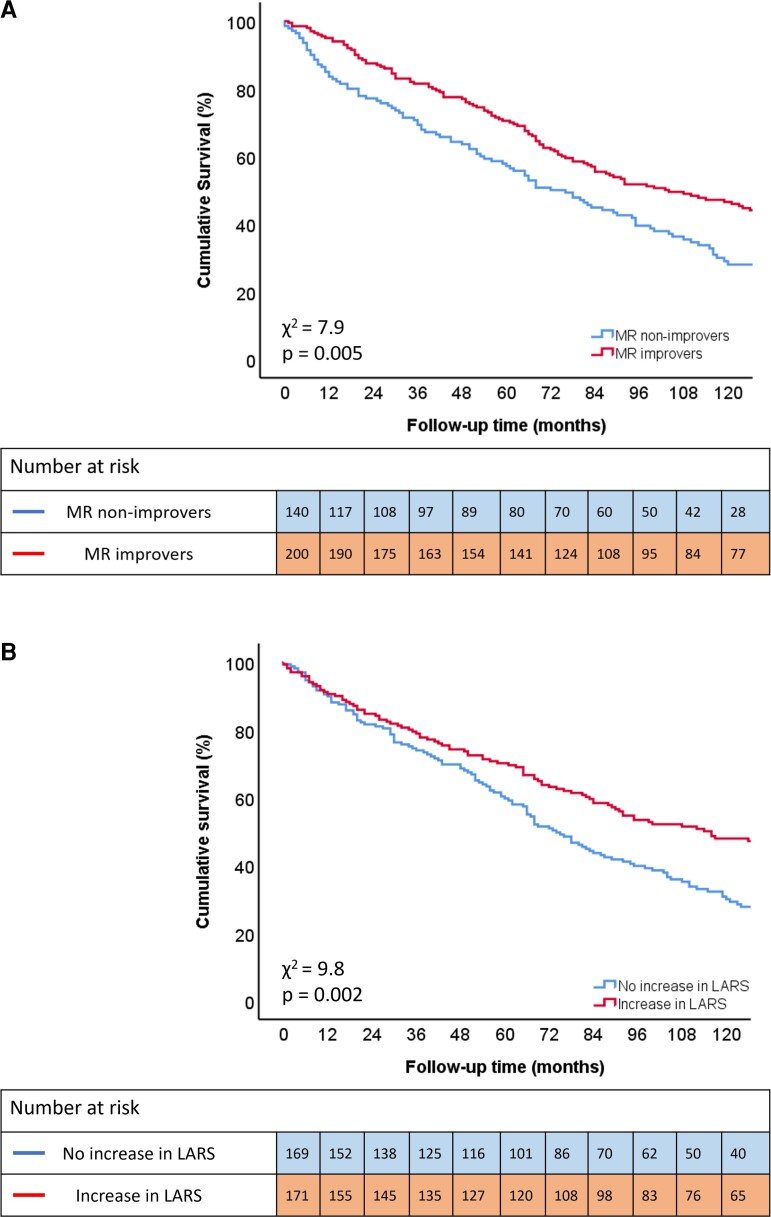
Kaplan–Meier curve for time to cumulative survival according to MR improvement (*A*) and increase in LARS (*B*).

During a median follow-up of 84 (39–135) months, 224 (66%) patients died. There was no significant difference in mortality rates between patients with moderate vs. severe MR at baseline (see Supplementary data online, *Figure S2*). In contrast, patients who had MR improvement experienced significantly lower mortality rates when compared to patients showing no MR improvement (5, 13, and 30% vs. 16, 23, and 43% at 1-, 2-, and 5-year follow-up, respectively, *P* = 0.005) (*Figure 1A*). There was no interaction between MR severity at baseline and MR improvement with outcomes (*P* = 0.526). Similarly, patients showing a greater increase in LARS (defined as a percentage change ≥9.4%, which is the median value of the relative change in LARS) experienced significantly lower mortality rates when compared to patients showing a smaller increase in LARS (defined as a percentage change <9.4%) (9, 15, and 30% vs. 10, 18, and 40% at 1-, 2-, and 5-year follow-up, respectively, *P* = 0.002) (*Figure 1B*). On multivariable Cox regression analysis, with LARS evaluated as a continuous variable, age (HR 1.032; 95% CI: 1.012–1.052; *P* = 0.002), serum creatinine (HR 1.008; 95% CI: 1.003–1.012; *P* < 0.001), baseline tricuspid annular plane systolic excursion (TAPSE) (HR 0.983; 95% CI: 0.970–0.997; *P* = 0.016), baseline peak tricuspid regurgitation velocity (HR 1.687; 95% CI: 1.211–2.350; *P* = 0.002) and relative change in LARS (HR 0.997; 95% CI: 0.995–1.000; *P* = 0.030) were independently associated with all-cause mortality, whereas MR improvement *per se* was not (HR 1.122; 95% CI: 0.788–1.596; *P* = 0.523) (*Table 5*). There was no interaction between atrial fibrillation and a relative change in LARS with outcomes (*P* = 0.678).

**Table 5:** Uni- and multivariable cox regression analyses to identify associates of all-cause mortality in patients with significant MR undergoing CRT implantation

	Univariable analysis		Multivariable analysis	
	HR (95% CI)	*P*-value	HR (95% CI)	*P*-value
Age, years	1.047 (1.032–1.063)	**<0.001**	1.032 (1.012–1.052)	**0.002**
Male sex	1.781 (1.289–2.460)	**<0.001**	0.790 (0.518–1.204)	0.273
eGFR, mL/min/1.73 m^2^	1.003 (1.002–1.004)	**<0.001**	1.008 (1.003–1.012)	**<0.001**
NYHA III–IV	1.551 (1.146–2.100)	**0.005**	1.136 (0.784–1.647)	0.500
Ischaemic aetiology	1.869 (1.411–2.474)	**<0.001**	1.285 (0.900–1.835)	0.167
Atrial fibrillation	1.088 (0.909–1.303)	0.356	—	
QRS duration, ms	1.000 (0.996–1.004)	0.833	—	
Baseline LV ESV, mL	1.001 (0.999–1.004)	0.181	—	
Relative change in LV ESV, %	1.016 (1.010–1.022)	**<0.001**	1.001 (0.992–1.011)	0.758
Baseline LVEF, %	0.991 (0.974–1.009)	0.322	—	
Relative change in LVEF, %	0.999 (0.998–1.001)	0.270	—	
Baseline LV GLS, %	0.947 (0.907–0.988)	**0.013**	0.977 (0.927–1.030)	0.383
Relative change in LV GLS, %	0.968 (0.953–0.984)	**<0.001**	0.996 (0.991–1.000)	0.077
Baseline TAPSE, mm	0.973 (0.963–0.984)	**<0.001**	0.983 (0.970–0.997)	**0.016**
Relative change in TAPSE, %	1.002 (0.992–1.012)	0.690	—	
Baseline TR max velocity, m/s	2.400 (1.786–3.223)	**<0.001**	1.687 (1.211–2.350)	**0.002**
Relative change in TR max velocity, %	0.994 (0.987–1.000)	0.060	—	
LAVi, mL/m^2^	1.014 (1.007–1.020)	**<0.001**	1.003 (0.994–1.012)	0.521
Relative change in LAVi, %	1.001 (0.998–1.005)	0.390	—	
LARS, %	0.959 (0.939–0.980)	**<0.001**	0.969 (0.937–1.002)	0.064
Relative change in LARS, %	0.997 (0.995–0.999)	**0.001**	0.997 (0.995–1.000)	**0.030**
MR improvement at 6 months	0.686 (0.527–0.894)	**0.005**	1.122 (0.788–1.596)	0.523

Values in bold have a *P*-value < 0.05.

ACE-i, angiotensin-converting enzyme inhibitor; ARB, angiotensin receptor blocker; BMI, body mass index; CABG, coronary artery bypass grafting; eGFR, estimated glomerular filtration rate; EDV, end-diastolic volume; ESV, end-systolic volume; LAVi, left atrial volume index; MRA, mineralocorticoid receptor antagonist; MWT, minute walking test; NYHA, New York Heart Association; PCI, percutaneous coronary intervention; QoL, quality of life; TR, tricuspid regurgitation.

**Figure 2:**
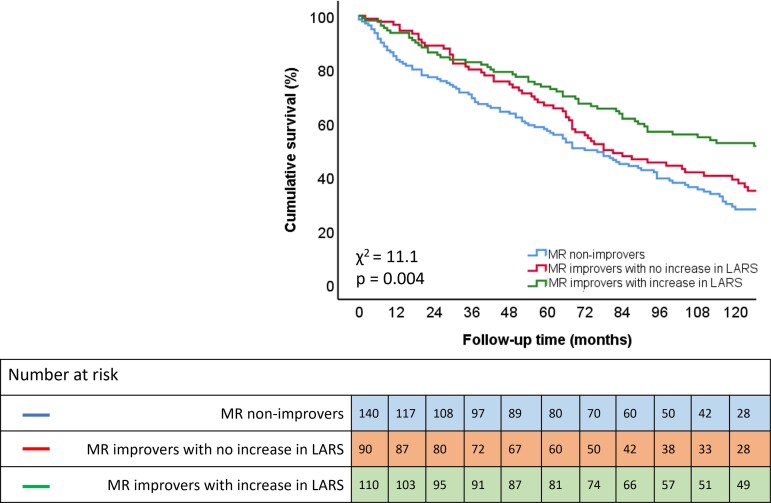
Kaplan–Meier curve for time to cumulative survival according to MR improvement and increase in LARS.

MR improvers who also experienced an increase in LARS, had significantly higher survival rates than MR improvers who did not experience an increase in LARS (*P* = 0.045) and MR non-improvers (*P* = 0.001). Outcomes were not significantly different between MR non-improvers and MR improvers showing no increase in LARS (*P* = 0.236) (*Figure 2*). The Kaplan-Meier curve according to an increase in LARS vs. no increase in LARS in patients without MR improvement, is shown in Supplementary data online, *Figure S3*.

## Discussion

The main findings of the current study can be summarized as follows: 1) a significant reduction in MR severity at 6 months after CRT implantation is independently associated with an increase in LARS, and 2) an increase in LARS is independently associated with lower all-cause mortality in patients with HF and significant functional MR after CRT implantation.

### Functional MR in CRT recipients

Functional MR is a common finding in patients with HF and reduced LVEF and is caused by progressive cardiac remodelling, resulting in papillary muscle displacement and annular enlargement.^18^ Previous studies have demonstrated that CRT may result in MR improvement,^19^ due to the immediate effects of resynchronization between the papillary muscles^20^ and the more delayed effects of LV reverse remodelling.^21^ In addition, an improvement in MR severity after CRT implantation has been associated with improved outcomes.^22^ The current study confirms these observations with 59% of the study population showing a significant improvement in MR severity (similar to the percentage mentioned in previous studies,^20^) which was associated with better prognosis. Although it has been clearly demonstrated that a reduction in functional MR after CRT translates into better outcome, the mechanisms underlying this benefit are still debated.

### LA function in CRT recipients

While research on post-CRT cardiac remodelling has mainly focused on the LV, beneficial effects may extend to the LA.^3,4^ HF causes a progressive reduction in LA compliance, which increases LA pressure and the haemodynamic stress on the pulmonary circulation, contributing to the development of postcapillary pulmonary hypertension and pulmonary vascular remodelling.^23^ In addition, LA adverse remodelling increases the risk of developing atrial fibrillation which is associated with an increased risk of cardiovascular events.^24^ LA remodelling has most often been defined as a change in LA volume, whereas LA function is rarely measured. Nonetheless, LARS (which is a functional parameter) has shown a good correlation with LA compliance and fibrosis on cardiac magnetic resonance imaging,^17^ and more accurately reflects acute changes in LV pressure than LA volume.^25^ In 405 patients with HF and reduced LVEF, Carluccio *et al*.^6^ demonstrated that LARS was a powerful predictor of outcomes, showing incremental prognostic information over LA volume and LV systolic function.

CRT has the ability to induce LA reverse remodelling, which has been associated with better cardiovascular outcomes.^10–12^ In a sub-study of the MADIT-CRT trial, a significant reduction in LA volume was seen after CRT implantation, which was associated with a reduced risk of atrial arrhythmias.^26^ Whether CRT can improve LA function (and therefore LA compliance) is less known. In a study of 30 patients undergoing CRT implantation, Valzania *et al*.^27^ showed an increase in LARS in CRT responders, whereas Dokuni *et al*.^28^ demonstrated an association between an increase in LARS after CRT and better clinical outcomes. There are different mechanisms that could contribute to the increase in LARS after CRT implantation. The major determinant of LA expansion is the systolic descent of the atrioventricular plane towards the apex, which is mainly driven by LV longitudinal function.^29^ CRT may increase LARS by reversing LV dyssychrony and improving LV longitudinal function. LARS is also associated with LV diastolic dysfunction, and an increase in LARS has been associated with an improvement in LV diastolic function after CRT implantation.^30,31^

### The interplay between functional MR and LA function

In patients with HF and reduced LVEF, LA afterload is increased due to an increase in LV end-diastolic pressure. Significant functional MR causes an additional volume overload on the LA, thereby accelerating LA adverse remodelling.^32^ Previous studies have demonstrated that functional MR severity is independently associated with LARS, with LARS in turn being associated with all-cause mortality and providing incremental prognostic value over LA volume.^7^ The current study expands on these results and for the first time shows that an improvement in functional MR severity after CRT implantation is independently associated with an increase in LARS. This increase in LARS was firmly linked to lower all-cause mortality. MR improvers showing a simultaneous increase in LARS had the lowest mortality rate, whereas outcomes were not significantly different between MR improvers showing no increase in LARS and MR non-improvers. Therefore, it appears that impaired LA dynamics not only reflect abnormalities in LV filling pressure (due to the underlying HF), but are also significantly influenced by the haemodynamic changes caused by the underlying functional MR. CRT may (partially) restore the impaired LA dynamics caused by functional MR, thereby improving prognosis. Interestingly, MR improvement was associated with an early survival benefit, but this was only maintained in patients who also showed an increase in LARS (*Figure 2*). MR improvement could perhaps be considered as a marker of improved haemodynamics (i.e. an immediate observed effect), whereas an increase in LARS could be considered a marker of LA reverse remodelling (i.e. a more long-term observed effect). Patients in whom LARS does not increase despite significant MR reduction, may have a more ‘proportionate MR’ (i.e. the degree of MR is expected for the amount of LV adverse remodelling and has no individual effect on LA dynamics) or the fibrotic LA may be irreversibly damaged. Interestingly, LARS was the only echocardiographic parameter in the current study in which a change after 6 months of CRT was associated with better outcomes. After extensively adjusting for various clinical and echocardiographic variables at baseline and follow-up, only a change in LARS, together with age, renal function, baseline RV systolic function and baseline peak pulmonary artery pressures were independently associated with long-term outcome.

### Clinical implications

Significant functional MR is strongly associated with a decreased quality of life and an increased risk of cardiovascular events, including HF hospitalization and mortality.^2^ Due to the high prevalence and poor outcomes, significant efforts have been undertaken to reduce functional MR severity and to understand which patients may benefit from interventional therapies. Although CRT has been shown to reduce functional MR, it remains incompletely understood how this improvement in functional MR benefits individual patients, in addition to the proven effects of CRT on LV reverse remodelling (independently of the presence of functional MR). It also remains incompletely understood why some patients with MR reduction after CRT do not derive any clinical benefit or reduction in cardiovascular events. Sequential measurements of LARS before and after CRT implantation may provide more insight into the beneficial effects of CRT and may allow the identification of a subgroup of patients who undergo significant functional MR improvement, but who will nevertheless experience suboptimal outcomes. The identification of such individuals may argue for more intensive follow-up, as the full benefit of MR reduction post-CRT cannot be expected in these patients. Whether more recently introduced HF treatments, such as sodium-glucose co-transport 2 inhibitors and angiotensin-neprilysin inhibitors may prove to have beneficial effects in these patients, requires further investigation.

### Limitations

This study is subject to the limitations associated with a retrospective, observational design. To include a large proportion of patients, a large time interval for patient enrollment was used. Data on changes in medical treatment during long-term follow-up were not available. LARS is a vendor-dependent parameter, and the values cannot be compared directly between different ultrasound platforms. RV systolic function was quantified with tricuspid annular plane systolic excursion, which is angle-dependent and does not account for regional differences in RV function. Although MR severity may be influenced by loading conditions, only haemodynamically stable patients were included in the present study. Quantitative measurements of MR severity were not provided. HF hospitalization, new onset atrial fibrillation or implantation of a LV assist device as an endpoint were not systematically recorded, and could not be analyzed. Moreover, the specific cause of death (cardiac or non-cardiac) was unknown in this study. However, it is less likely that CRT induced LA remodelling at 6 months after CRT would affect non-cardiovascular mortality.

## Conclusion

In patients with HF and significant functional MR, an improvement in MR after CRT implantation is independently associated with an increase in LARS, which in turn, is associated with better survival.

## Supplementary Material

jeac149_Supplementary_Data

## Data Availability

The data underlying this article will be shared on reasonable request to the corresponding author.
