# Supplementary figures and images for: Interaction between secondary mitral regurgitation and left atrial function and their prognostic implications after cardiac resynchronization therapy
Source: Eur Heart J Cardiovasc Imaging. 2022 Jul 28;24(4):532–41. doi: 10.1093/ehjci/jeac149 (PMC10029846; doi:10.1093/ehjci/jeac149)

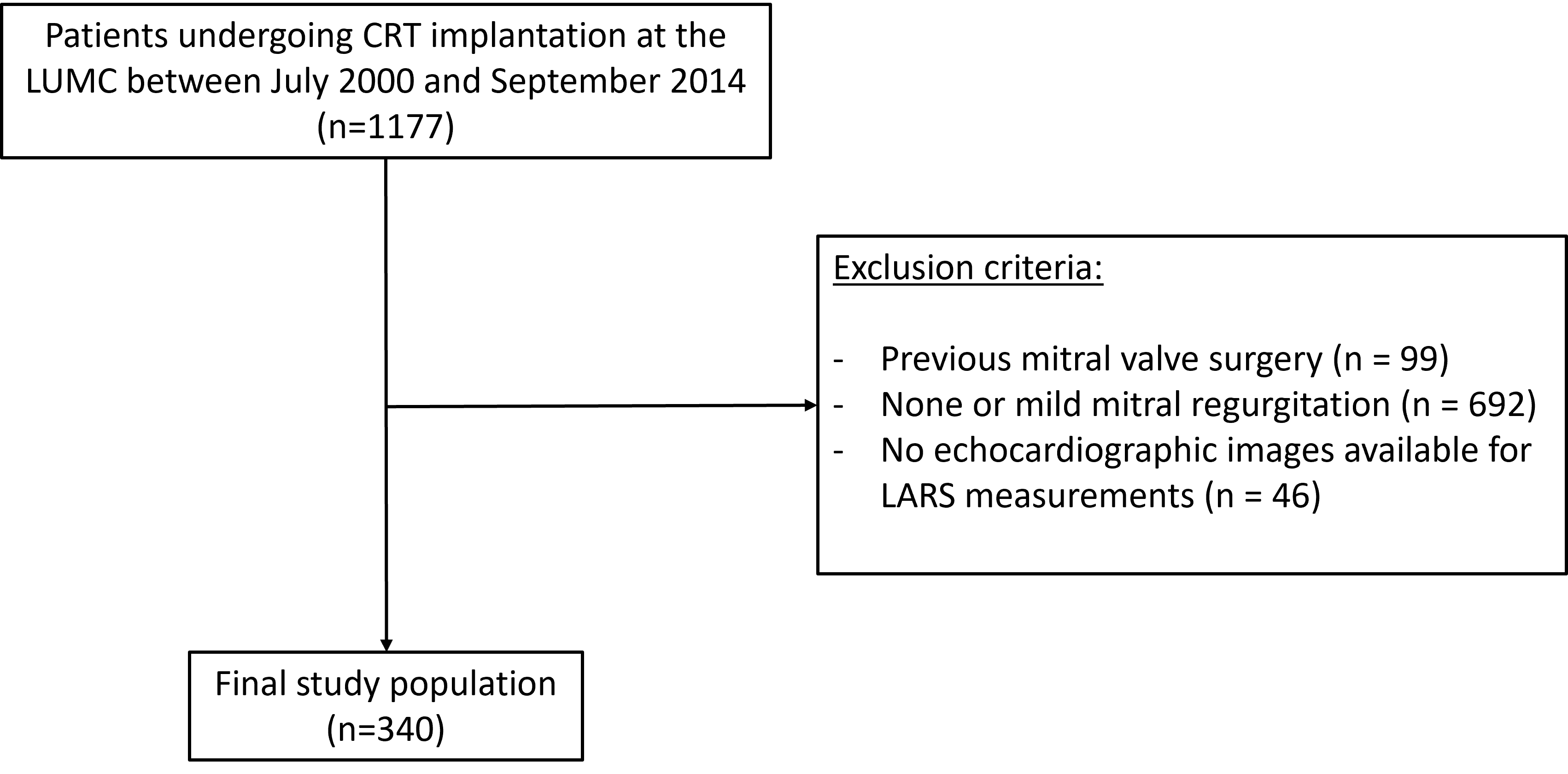

Supplement: jeac149_Supplementary_Data [file jeac149_supplementary_data.zip › Figure_S1.tif]

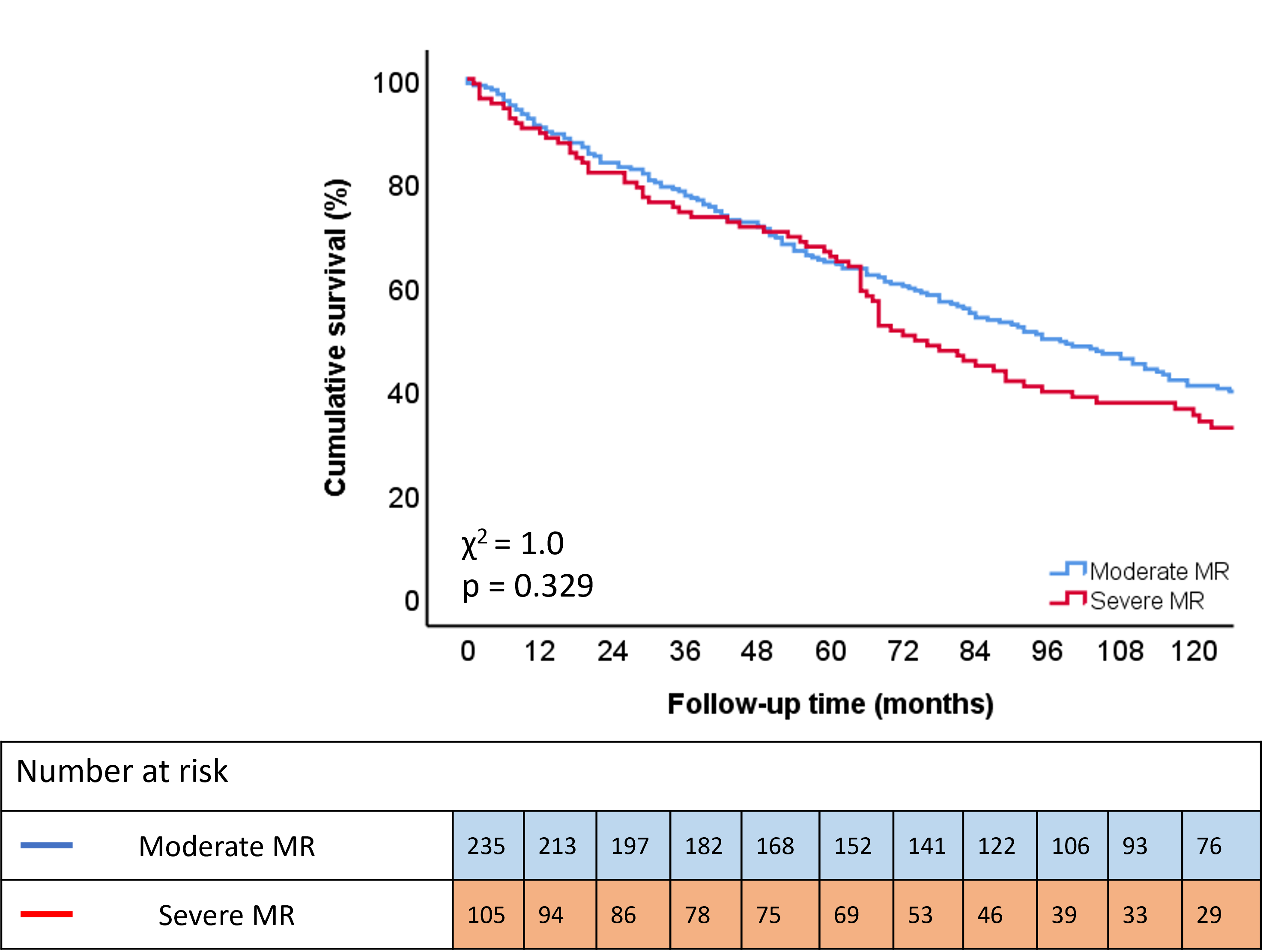

Supplement: jeac149_Supplementary_Data [file jeac149_supplementary_data.zip › Figure_S2.tif]

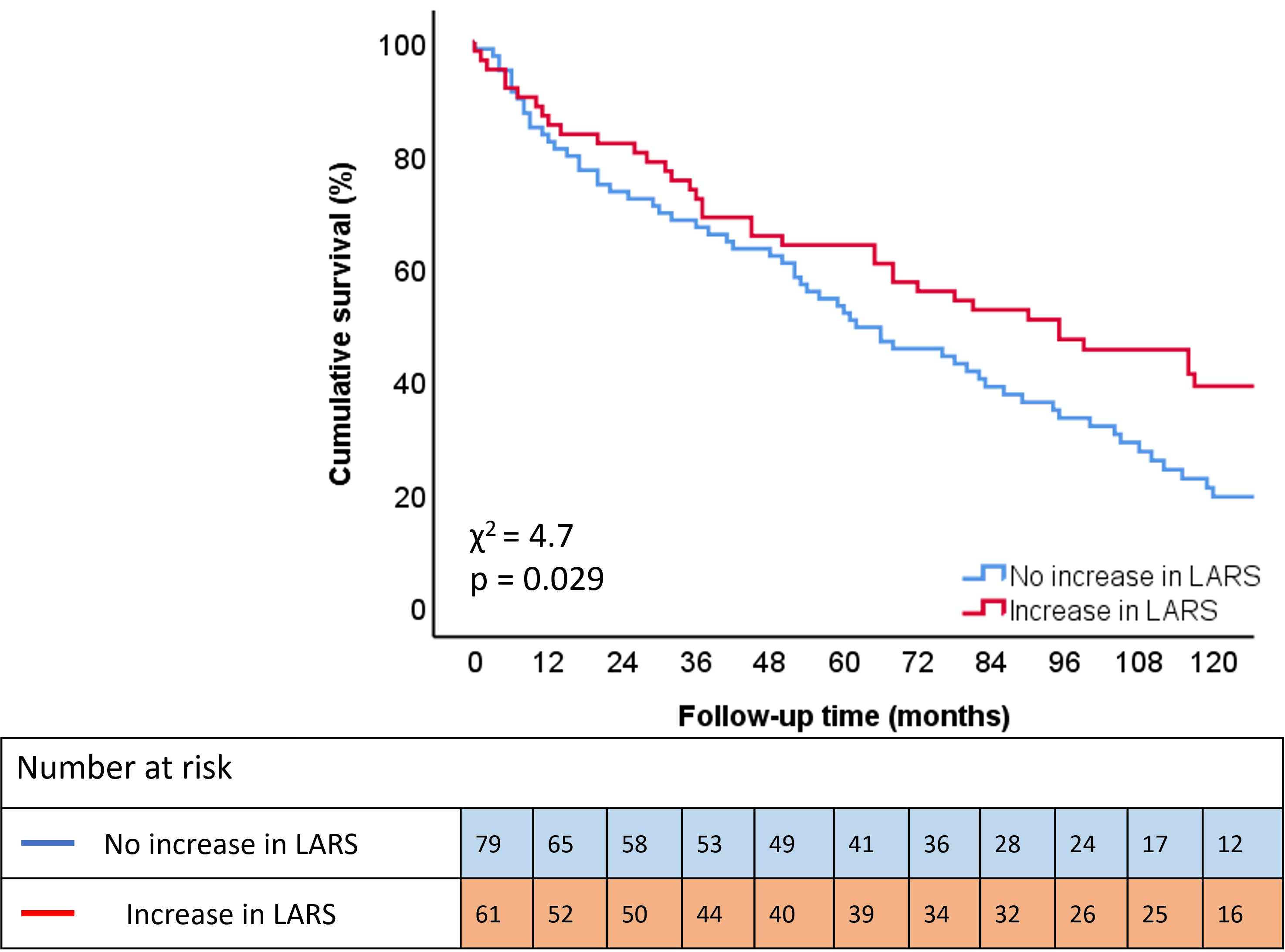

Supplement: jeac149_Supplementary_Data [file jeac149_supplementary_data.zip › Figure_S3.tif]
